# O041. GRIA3 (glutamate receptor, ionotropic, ampa 3) gene polymorphism influences cortical response to somatosensory stimulation in medication-overuse headache (MOH) patients

**DOI:** 10.1186/1129-2377-16-S1-A49

**Published:** 2015-09-28

**Authors:** Cherubino Di Lorenzo, Gianluca Coppola, Gaetano Grieco, Filippo Maria Santorelli, Esterina Pascale, Francesco Pierelli

**Affiliations:** 1 Don Carlo Gnocchi Onlus Foundation, Rome, Italy; 2 Department of Neurophysiology of Vision and Neuro-ophthalmology, G. B. Bietti Foundation-IRCCS, Rome, Italy; 3 IRCCS Neurological Institute “C. Mondino”, Laboratory of Neurogenetics, Pavia, Italy; 4 IRCCS Fondazione Stella Maris, Molecular Medicine & Neurodegenerative Diseases, Pisa, Italy; 5 Department of medico-surgical sciences and biotechnologies, “Sapienza" University of Rome, Rome, Italy; 6 IRCCS Neuromed, Pozzilli, IS Italy

## Introduction

Medication-overuse headache (MOH) is a secondary form of chronic headache developed by migraineurs after prolonged symptomatic medication overuse. Bio-behavioural sensitization is a key mechanism in MOH pathophysiology, as evidenced by cortical somatosensory evoked potentials (SSEPs) studies. While episodic migraineurs, recorded between attacks, showed lower initial SSEP amplitudes and lack of habitation during stimulus repetition, in MOH patients the SSEPs were initially higher and further increased during stimulus repetition, resulting in a persistent sensitisation proportional to the duration of the headache chronification phase. The central sensitization seems to be strongly dependent by glutamate. Amongst various gene polymorphisms in the glutamatergic system, only the Glutamate Receptor Ionotropic AMPA 3 (GRIA3) was previously associated with migraine. The aim of our study was to verify whether GRIA3 rs3761555 single nucleotide polymorphism (SNP) could influence processes of central sensitization of MOH patients.

## Methods

We measured SSEP amplitudes as a marker of sensitization, and SSEP habituation over two sequential blocks during uninterrupted peripheral stimulation in a well-characterized group of 60 MOH patients who underwent GRIA3 rs3761555 polymorphism analysis.

## Results

Sixty (47 females) MOH patients were enrolled in the study: 27 (9 males) resulted as T/T and 26 C/T and 7 (4 males) T/T. In the comparison among the three genotypes, the grand average of all the neurophysiological data did not emerge in terms of latencies and amplitudes. The analysis of block amplitude averages showed differences in SSEP 1^st^ (p = 0.028) and 3^rd^ (p = 0.023) block amplitude.

## Discussion

Our findings are consistent with the hypothesis that the glutamatergic system influences central sensitization processes in MOH patients, by plastic changes in the “pain matrix”, resulting in decreased nociceptive thresholds, increased responsiveness to peripheral stimuli and expansion of the receptive fields of central nociceptors. These phenomena are at the base of migraine chronification, maybe due to the higher levels of glutamate, as it is measured in the CSF of chronic migraineurs. Indeed, we observed that although MOH patients overall had notoriously larger SSEP 1^st^ block amplitude than controls, and deficient habituation, GRIA3 rs3761555 SNP influenced the amplitude of blocks, according to a decreasing gradient from T/T to C/C subjects. Although the analyzed SNP functional consequences are unknown, it was highlighted as somehow implicated in migraine pathophysiology in two independent cohorts of patients, maybe by an altered transcriptional activity. Hitherto, we are not aware of other disorders potentially related to this SNP.

Written informed consent to publication was obtained from the patient(s).

